# Cancer mortality in a Chinese population surrounding a multi-metal sulphide mine in Guangdong province: an ecologic study

**DOI:** 10.1186/1471-2458-11-319

**Published:** 2011-05-16

**Authors:** Mao Wang, Hong Song, Wei-Qing Chen, Ciyong Lu, Qianshen Hu, Zefang Ren, Yan Yang, Yanjun Xu, Aiming Zhong, Wenhua Ling

**Affiliations:** 1 Department of Preventive Medicine, School of Public Health, Sun Yat-Sen University, Guangzhou, China; 2 Department of Medical Statistics and Epidemiology, School of Public Health, Sun Yat-Sen University, Guangzhou, China; 3 The Centre for Disease Control and Prevention of Guangdong province, Guangzhou 510300, China; 4 The Centre for Disease Control and Prevention of Wengyuan County, Shaoguan 512600, China; 5 Department of Nutrition, School of Public Health, Sun Yat-Sen University, Guangzhou 510080, China

## Abstract

**Background:**

The Dabaoshan mine in the southeast of Guangdong Province, China, is at high risk of multi-metal pollutant discharge into a local river (Hengshihe) and the surrounding area. Following approximately 30 years of exposure to these metals, little is known regarding the subsequent health effects and risks for the local residents. In our present study, we have estimated the relationships between long-term environmental exposure to multiple heavy metals and the risk of cancer mortality in a Chinese population in the vicinity of Dabaoshan.

**Methods:**

An ecologic study was performed. Between 2000-2007, a total population of 194,131 lived in the nine agricultural villages that surround the Hengshihe area. Heavy metals concentrations were determined in local environmental samples (water and crops) and whole blood taken from 1152 local residents of both a high-exposure area (HEA) and a low-exposure area (LEA). We calculated the rate ratio and standardized mortality ratios based on age- and gender-specific cancer mortality rates for the different reference populations (based on district, county and province). Simple, multiple linear and ridge regression models were used to evaluate the associations between exposure to multiple heavy metals and cancer mortality in the nine villages, after adjustment for age and sex.

**Results:**

The geometric mean blood levels of cadmium and lead were measured at 24.10 μg/L and 38.91 μg/dL for subjects (n = 563) in the HEA and 1.87 μg/L and 4.46 μg/dL for subjects (n = 589) from the LEA, respectively (*P *< 0.001). The rate of mortality from all cancers in the HEA was substantially elevated in comparison with the corresponding mortality rate in the LEA for men (rate ratio = 2.13; 95% confidence intervals = 1.63 - 2.77) and women (2.83; 1.91 - 4.19); rates were also significantly elevated compared with the rate when compared to the entire Wengyuan County area, or the provincial reference population. In addition, mortality rates were significantly increased for stomach, lung and esophageal cancer in the HEA in comparison with the corresponding rates in the LEA, in Wengyuan County and the provincial reference population for men, women and both combined. Further analysis showed that there were significantly positive correlations between exposure to cadmium and lead and the risk of all-cancers and stomach cancer mortality among women and both sexes, whilst zinc exposure showed no association with the risk of site-specific cancer mortality in the nine villages evaluated.

**Conclusions:**

The findings of this study reveal probable associations between long-term environmental exposure to both cadmium and lead and an increased risk of mortality from all cancer, as well as from stomach, esophageal and lung-cancers.

## Background

The Dabaoshan mine, built in 1958, is located to the southeast of Shaoguan City, Guangdong Province, China. Since the 1970s, the mine has been in full-scale operation as a large-scale and integrative quarrying mine. This facility plays an important role for both the non-ferrous metal materials and steel industries in southern China. The mine itself contains multi-metal sulphide mineral deposits, including limonite in the superior part of ore body with a reservoir of 20 million tons, and copper-sulphide lying in the inferior part of the ore body with a reservoir also of 20 million tons [1]. Mineral separation at the mine has been incomplete, and most of the lean ore has been discarded and become gradually weathered. During processing, including leaching, ore dressing, and washing, substantial quantities of waste water are discharged directly into the environment. Both the discarded ore and waste water from the mine constitute severe environmental pollutants for the surrounding and downstream areas.

Previous surveys [2,3] have shown that the pH of the wastewater from Dabaoshan is 2.15, and that the cadmium (Cd), lead (Pb) and zinc (Zn) concentrations in the irrigation water of nearby crop regions were 16-, 5.0- and 3-fold higher, respectively, than the government approved standards (The Standards for Irrigation Water Quality, GB 5084-1992). Zhou et al. [1] have further found that the pH of the soils in the fields irrigated with this polluted water was approximately 4.0, and that the lead and cadmium concentrations in the soil are 44-fold and 12-fold higher, respectively, than the government standards (The Environmental Quality Standard for Soils, GB 15618-1995). One study in 2005 has also reported that the concentrations of heavy metals in soils in the Hengshihe River region exceed the national standards (China), particularly for Zn, copper (Cu) and Cd [4]. The definition of a "heavy metal" includes a specific gravity of more than 5 or a density greater than 4.5 g/cm^3^, and the 45 established heavy metals include Cu, Zn, Pb, Cd and manganese [5]. A previous study by Cai et al. [6] has reported that the levels of Pb, Zn, Cu and Cd in sediment from the Hengshihe river, which functions as a mine drainage afflux, are as high as 1841.02, 2326.28, 1522.61 and 10.33 mg/kg respectively. These levels are far higher than the maximum concentrations recommended for soil, in particular the Cu and Cd concentrations which were found to be 14-fold and 4-fold higher, respectively, than the government standards (GB 15618-1995) [6]. The surface-layer soils in the coastal areas near to the Hengshihe river also showed large aggregations of Cd, Pb, Zn and Cu.

Cadmium and cadmium compounds have been established as carcinogens, mainly from epidemiological data for occupational exposure [7], whereas lead is classified as a possible carcinogen in humans [8,9]. However, many previous epidemiologic studies of the relationships between Cd or Pb exposure and cancer have only been undertaken in occupational settings [10,11]. There is strong evidence for a positive association between occupational exposure to Cd and lung cancer risk [10,12]. However, the association of Cd with other potential target organs, such as the stomach, liver and prostate, remains equivocal [13-15]. Moreover, although both of these heavy metals are ubiquitous environmental pollutants, there is a current lack of evidence for human cancer causation by oral exposure to Cd or Pb [15,16]. In addition, due to the antioxidant role of zinc, it is conceivable that there may be a protective effect of a high zinc status against cancer development [10,17].

In addition to Cd, Pb and Zn, Cu is another environmental pollutant from Dabaoshan, which contains substantial copper-sulphide mineral deposits. Cu is an essential nutrient and a redox-active transition metal that may initiate oxidative damage to lipids, proteins, DNA and contribute to neurodegenerative disorders, especially at high concentration [18]. High levels of Cu can also induce growth proliferation and cancer by damaging DNA via toxic free hydroxyl radicals [19]. Chronic Cu toxicity is rare and primarily affects the liver [20]. On the other hand, Cu is essential for optimal antioxidant defense, and a Cu deficiency may increase cellular susceptibility to oxidative damage [21]. High intakes of ascorbic acid and zinc may reduce Cu toxicity by reducing Cu intake [22,23]. To our knowledge however, there have been few studies of the associations between environmental exposure to multiple heavy metals over a period as long as 30 years and human cancer mortality.

Because the Hengshihe river is the main drainage pathway for effluent from the Dabaoshan mine, it has been polluted since the 1970s. The river thus delivers significant quantities of heavy metals to numerous villages in its region. After about 30 years of exposure to these metals, some local residents in the Hengshihe area have begun to acquire upper gastrointestinal diseases, for example, both oesophageal and stomach cancer are of a priori interest. To date however, there has been no study of the detrimental effects of long-term environmental exposure to multiple heavy metals on the local general population other than our previous report that heavy metal exposures are associated with an increased risk of behavioral problems in school-aged children [24].

In our current study, we aimed to determine the heavy metal levels in the water, supply, field crops and residents' blood in the area around Dabaoshan mine and evaluate the relationships between these exposures and the human cancer mortality rates.

## Methods

### Study area and population

**Figure 1:**
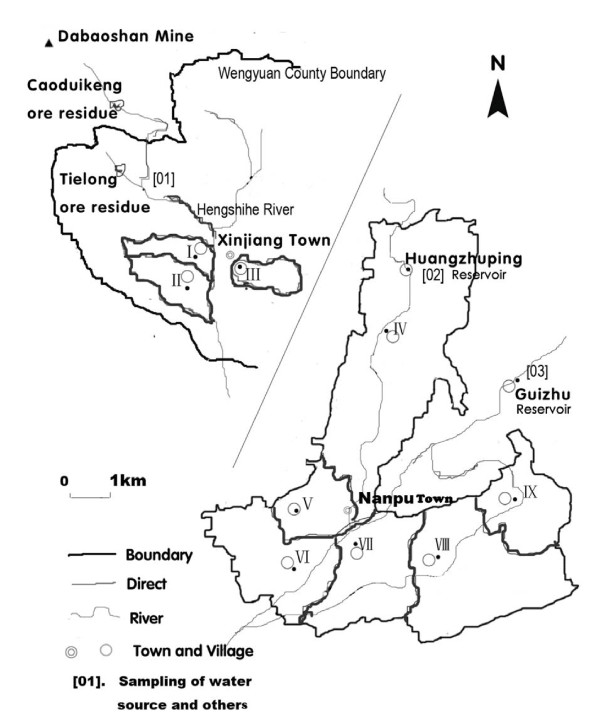
**Geographic map of the region encompassing the Dabaoshan Mine (▲), the surrounding area, the HEA [Shangba Village (I), Xiaozhen Village (II) and Dongfang Village (III)] and LEA [Zhongxin Village (IV), Shaping Village (V), Shuikou Village (VI), Fengshan Village (VII), Mashan Village (VIII) and Madun Village (IX)]**.

The present study was conducted in a high-exposure area [HEA] downstream of the Hengshihe river in Shaoguan city, including the villages of Shangba (I), Xiaozhen (II) and Dongfang (III), and a low-exposure area [LEA] which was selected as a reference area about 35 kilometers from the river and includes the villages of Zhongxin (IV), Shaping (V), Shuikou (VI), Mashan (VII), Fengshan (VIII), and Madun (IX) (Figure 1). The Hengshihe river is the source of drinking and irrigation water for Shangba and Xiaozhen, and ground water, which may be contaminated by Hengshihe, is the source of drinking and irrigation water for Dongfang village. In the LEA, the sources of drinking and irrigation water are the Huangzhuping and Guizhu reservoirs. The nine villages under study belong to areas of Wengyuan County and most (>95%) of the residents are moderately educated farmers with an average annual income per capita of less than US$556.00. There is no heavy industry in this region and little air pollution from automobiles. The two reference populations comprised individuals who lived in Wengyuan County and the rural areas of Guangdong province (RAGDP) from 2004-2005 [25].

### Water, soil and crop analysis

Water, soil and crop samples were taken from both the HEA and LEA using standard methods as previously described [24]. One and two water source samples, respectively, were taken from the HEA and LEA. Irrigation water, well, soil, rice, and avena nuda samples were also taken from each village. The levels of Cd, Pb, Cu and Zn in these environmental samples were determined by standard methods as previously described [24].

### Blood analysis

A cross-sectional study was performed in July 2006. The samples were selected using simple random sampling. In each village, we identified a random population sample stratified by sex and age (18-39 years *vs *40-59 years *vs *60-74 years *vs *≥75 years), with the aim of recruiting equal numbers from each group. The nine village municipalities gave listings of all inhabitants sorted by address. Households, defined as those who lived at the same address, were used as the sampling unit. We numbered the households consecutively, and generated a random-number list using the SPSS random function. Households with a number matching the list were invited to participate and any household member older than 18 years was eligible. Individuals were not included if the quota for age-sex stratum had been met. Among a total of 1180 eligible participants, 1152 (97.6%) completed the survey. The study population thus comprised 563 residents from the HEA and 589 from the LEA. At baseline, we used a validated questionnaire that asked about lifestyle, consumption of local food including rice, water and various crops, past and current place of residence, possible exposures to toxic substances, smoking and drinking habits, and previous medical history. Whole blood samples were collected from all 1152 participants by well-trained nurses and shipped at -20°C to our laboratory at the School of Public Health, Sun Yat-Sen University in an appropriate manner to avoid any external contamination.

Microwave-assisted acid digestion was conducted using previously described methods for whole blood [24,26]. The accuracy of the results was evaluated using a cattle serum (freeze-dried) reference sample (Chinese Certificated Reference Materials GBW(E) 090006, Beijing, China). Atomic absorption measurements were made using a polarized Zeeman atomic absorption spectrometer (Hitachi-5000, Japan). The detection limits were set at 0.8 μg/L for Pb, 0.06 μg/L for Cd, 0.5 μg/L for Cu and 0.005 mg/L for Zn. The precision of the measurements was in the range of ± 3-5%.

### Retrospective ecological mortality studies

A third retrospective cause of death investigation of residents living in the Guangdong province from 2004-2005 was conducted in August 2006 [25]. The investigation samples were selected using a proportional allocation stratified cluster random sampling design. According to the stratification methodology of the National Bureau of Statistics of China, cities and counties/districts belonging to Guangdong province are classified as either big cities (population >500,000), middle and small cities (population <50,0000), I rural areas and II rural areas [27]. Using the data from the third National Census, rural areas are classified as richer, rich, poor and poorer according to health status (mortality, infant mortality and birth rate), population structure (number of people younger than 14 and older than 65 in the population and the population density), and economic conditions (the per-capita gross output values from the industry and agriculture sectors, activity rate for secondary industries, and the illiteracy rate). The richer and rich areas are classed as I rural whereas poor and the poorer areas are classed as II rural [27].

**Figure 2:**
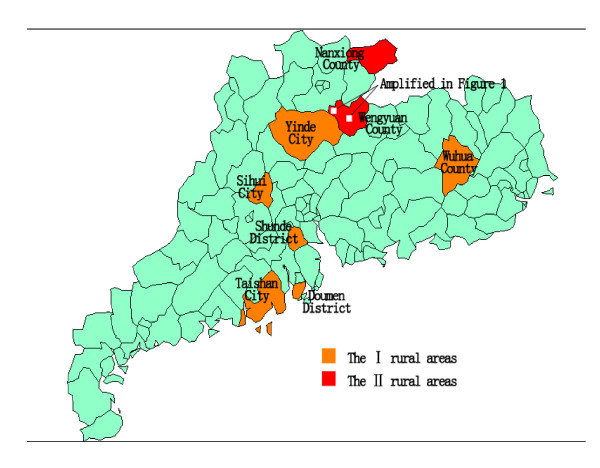
**Geographic map of the third retrospective investigation of causes of death in the RAGDP of China spanning 2004-2005 conducted by the provincial health department and the School of Public Health of Sun Yat-Sen University (Notes: These areas were classified as type I or type II rural areas according to the socioeconomic status (SES)**. Type I rural areas had a high SES and included Shunde district, Sihui city, Wuhua county, Doumen district, Taishan city and Yingde city. The type II rural areas had a low SES and included Wengyuan county and Nanxiong county.

The definition of proportional allocation is the proportion of each of these four stratifications within the sample cohort that is coincident with that of Guangdong province according to the fifth National Census in China in 2000 (1.3: 1.8: 7.2: 1) [28]. Concurrently, we performed the same cause of death survey for the period 2000-2007 in the nine villages included in our present report. The residents living in Wengyuan County and in the RAGDP from 2004 to 2005 from the third retrospective investigation were selected as our two reference populations (Figure 2). Considering the different environmental and lifestyle factors underlying cancer, the residents living in the town where the county government was located were excluded from the RAGDP population. All reported deaths in the above-studied population had death certificates archived by local police departments, 89% of which were confirmed by a county physician [29]. Moreover, all death certificates were verified through door-to-door surveys or by the well-trained investigators at the local examination centers. To control for quality of the analyses, strict measures were used for every component of the investigation, including the design stage, on-the-spot investigations, data collection, sorting and analysis [30]. Twelve trained nosologists had previously validated the coding used on the death certificates [31]. The *International Classification of Diseases*, tenth revision (ICD-10) [32] was used to identify deaths that were due to a malignant neoplasm (codes C00-C97) [33].

### Statistical analysis

The heavy metal concentrations in various sample types from the HEA were compared with counterpart samples from the LEA and with the current government standards in China. Log transformations were used to adjust the blood metal levels and age-adjusted cancer mortality rates for skewness. Blood metal levels were reported as the geometric mean and standard deviation (SD). We compared mean values using the standardized normal *z *test, and frequencies using chi-square test. Data from the Fifth National Census in China in 2000 for gender and 5-year age groups were used to calculate both the standardized mortality rate and expected number of cancer deaths in each of nine studied villages, the HEA, the LEA, Wengyuan County and the RAGDP population.

For comparison of the average cancer mortality rates in the HEA with those in the LEA, a binomial statistical distribution was assumed for both data sets when performing chi-square tests to calculate association probabilities for the ratio of rates (RR). This was also assumed when using 95% confidence intervals and 2-sided hypothesis test probabilities with the PEPI Compare2 program using the option "Rates with no.-of-individuals denominators" [34,35] for the comparison of the rates in the HEA to the averaged rates in Wengyuan County and the RAGDP population from 2004-2005, respectively (the middle of 2000-2007 survey period). A Poisson statistical distribution was assumed in calculating the exact mid-P 95% CIs and 2-sided hypothesis test probabilities for 70 or fewer deaths, and approximate Fisher CIs and probabilities for more than 70 deaths, using the PEPI Describe computer program with the option "Compute SMR or indirectly standardized rate" [34,35].

At the population level, simple, multiple linear and ridge regression models were used to further analyze correlations between blood heavy metal concentrations and age-adjusted mortality from selected site-specific cancer in the nine villages evaluated. If the blood heavy metal concentrations were found to be highly correlated with each other, then collinearity was considered as it is well known that it can affect the accuracy of the results [36]. Hence, a ridge regression was applied to address the instability of parameter estimates in the presence of collinearity, adjusting for gender and age. Ridge regression models were developed based on the adjusted R^2 ^selection method. Ridge regression stabilizes model parameter estimates by multiplying a contant (i.e., Ridge *k*) to the elements of the correlation matrix, displacing it from singularity and statistically compensating for the effects of multicollinearity. Ridge Trace and variance inflation factor (VIF) plots were employed to determine the most efficient Ridge *k *used to bias least-square estimators of model parameters (i.e., coefficients), because increasing values of the Ridge *k *also superficially inflate the mean square error of the model, which has negative consequence on model diagnostics. The objective was to determine the lowest Ridge *k *that reasonably stabilized the coefficient and their VIFs, which reflect the variance of a coefficient relative to its variance if all the predictor variables in the model were uncorrelated. All analyses were carried out separately for men and women. All statistical analyses were performed using SPSS software, version 13.0 (SPSS Inc., Chicago, IL). All significance tests were two-sided using 0.05 as the level of statistical significance.

### Ethical approval

This study received approval from the Sun Yat-Sen University, School of Public Health Ethics Committee. All participants provided signed informed consent.

## Results

### Environmental pollution

The heavy metal concentrations in environmental samples from the HEA were found to be far higher than those from the LEA and the accepted government standards (Table 1). In the HEA, the waste water was acidic (pH = 3.35) and contained concentrations of cadmium and zinc that reached 7.09 × 10^-3 ^mg/L and 13.7 mg/L, respectively, which far exceeded the government standards of 5.00 × 10^-3 ^mg/L and 1.00 mg/L, respectively. A significantly higher content of both Cd and Zn than allowable by government standards were also found in the irrigation water and wells in Shangba village, which is the closest population center to the Dabaoshan mine. In the village of Dongfang, the Pb concentration in the avena nuda, soil and rice samples was higher than the corresponding levels in the LEA. The highest concentrations of Cd and Pb were measured at 0.47 mg/kg (Shangba), and 830 mg/kg (Shangba), respectively, in rice; and 0.13 mg/kg (Shangba), and 1690 mg/kg (Xiaozhen), respectively, in avena nuda.

**Table 1:** Heavy Metal Concentrations in Environmental Samples near a Multi-metals Sulphide Mine in Guangdong, China

	High-Exposure Area			Low-Exposure Area	**GS **^**a**^
			
	Shangba	Xiaozhen	Dongfang	Six villages	
N (excluding water source)	1	1	1	6	
pH					
Water source	3.35 (Wastewater) ^b^			7.10 (Huangzhuping) 7.12 (Guizhu) ^c^	6.0-9.0
Irrigation water	4.92	7.75	7.76	7.02 - 7.35	5.50-8.50
Well water	4.77	7.06	6.88	7.04 - 7.56	6.5-8.5
Cadmium					
Water source (mg/L,×10^-3^)	7.09 (Wastewater) ^b^			0.09 (Huangzhuping) 0.10 (Guizhu) ^c^	5
Irrigation water (mg/L,×10^-3^)	8.31	4.25	0.66	<0.001	5
Well water (mg/L, ×10^-3^)	8.88	0.13	0.07	<0.001	5
Soil (mg/kg)	0.528	0.422	0.043	0.032 - 0.18	0.30
Rice (mg/kg)	0.47	0.36	0.02	0.006 - 0.075	0.20
Avena nula (mg/kg)	0.13	0.06	0.01	0.004 - 0.060	0.20
Lead					
Water source (mg/L)	0.043 (Waste water) ^b^			0.01 (Huangzhuping) 0.01 (Guizhu) ^c^	0.010
Irrigation water (mg/L)	0.045	0.035	0.013	<0.01	0.200
Well water (mg/L)	0.012	0.017	0.016	<0.01 - 0.02	0.01
Soil (mg/kg)	600	770	68	7.13 - 19.93	300
Rice (mg/kg)	830	520	280	<0.05	300
Avena nula (mg/kg)	820	1690	1050	0.030 - 0.145	300
Copper					
Water source (mg/L)	0.196 (Wastewater) ^b^			0.01 (Huangzhuping) 0.01 (Guizhu) ^c^	1.000
Irrigation water (mg/L)	0.631	0.020	0.020	<0.01	0.500
Well water (mg/L)	1.570	0.020	0.045	<0.01	1.00
Soil (mg/kg)	1261	147	20	2.85 - 8.19	100
Rice (mg/kg)	5.38	3.08	2.03	0.43 - 0.69	10
Avena nula (mg/kg)	1.31	0.82	0.42	0.23 - 1.09	- ^d^
Zinc					
Water source (mg/L)	13.700 (Wastewater) ^b^			0.01 (Huangzhuping) 0.01 (Guizhu) ^c^	1.000
Irrigation water (mg/L)	25.200	0.048	0.005	<0.01 - 0.84	2.00
Well water (mg/L)	4.89	0.024	0.011	<0.01 - 0.49	1.00
Soil (mg/kg)	680	227	68	2.85 - 8.19	250
Rice (mg/kg)	29.40	20.70	13.80	7.50 - 10.90	50
Avena nula (mg/kg)	20.80	5.12	3.73	2.89 - 7.94	- ^d^

^a ^Government standard.

^b ^Sampling point of wastewater [01] in the high-exposure area (see **Figure 1**).

^c ^Sampling points of water source [02] and [03] in the low-exposure area (see **Figure 1**).

^d ^No government standard.

### Social-demographic characteristics and heavy metal levels in blood

A cross-sectional study revealed that there were similar socioeconomic status (SES), dietary and geographic characteristics among the nine studied villages. Our analysis showed that the percentage of residents consuming local produce among the HEA participants (n = 563) was 96.1% and LEA participants (n = 589) was 98.3%. The consumption frequency of produce grown on local farms situated in the HEA is significantly lower than that in the LEA. There was no difference in the rate of cigarette smoking and alcohol consumption, and none of the women living in these villages had a smoking habit (data not shown).

Based on the dwelling time of the HEA participants (n = 563), the median duration of exposure to pollutants from Dabaoshan mine was 28 years. The geometric mean blood levels of Cd, Pb, Cu and Zn in subjects from the HEA were measured at 24.10 μg/L, 38.91 μg/dL, 0.82 mg/L and 8.38 mg/L, respectively (Table 2). The numbers of blood Cd and Pb levels of 1152 residents living in the HEA and LEA were represented in Additional file 1, 2, 3 and 4, figure s1-s4. The Cd and Pb levels were higher in the older subjects and highest in former smokers and alcohol drinkers. The mean blood concentrations of Cd, Pb and Zn were also significantly elevated in comparison with the levels in the LEA residents, although the mean blood Cu concentrations were not significantly different among the two populations (See Additional file 5: table s1).

**Table 2:** Blood Levels of Cadmium, Lead, Copper and Zinc by Participant Characteristics

		Geometric mean (SD)			
		
Characteristics	n	Cadmium, ug/L	Lead, μg/dL	Copper, mg/L	Zinc, mg/L
The high-exposure area (H) (I-III)	563	24.10 (3.52) ^a^	38.91 (0.39) ^a^	0.82 (1.49)	8.38 (1.96) ^a^
Shangba (I)	198	34.80 (2.96)	67.36 (0.24)	0.84 (1.42)	11.23 (1.75)
Xiaozhen(II)	177	10.33 (4.16)	10.35 (0.40)	0.73 (1.26)	5.52 (1.45)
Dongfang (III)	188	21.37 (3.11)	62.55 (0.28)	0.86 (1.71)	8.94 (2.09)
The low-exposure area (L) (IV-IX)	589	1.87 (2.48) ^a^	4.46 (0.18) ^a^	0.81 (1.24)	7.96 (1.32) ^a^
Zhongxin (IV)	172	1.69 (2.34)	5.81 (0.17)	0.86 (1.27)	8.47 (1.34)
Shaping (V)	72	2.06 (2.57)	4.70 (0.18)	0.81 (1.16)	7.80 (1.30)
Shuikou (VI)	72	1.90 (2.51)	3.79 (0.15)	0.85 (1.29)	8.34 (1.19)
Fengshan (VII)	104	1.23 (2.45)	4.34 (0.18)	0.79 (1.14)	8.17 (1.24)
Mashan (VIII)	86	2.15 (2.12)	2.97 (0.19)	0.72 (1.19)	7.51 (1.27)
Madun (IX)	83	3.03 (2.40)	4.47 (0.15)	0.76 (1.25)	7.01 (1.45)
Age, yrs					
18-39					
H	106	24.47 (3.15)	39.98 (0.42)	0.78 (1.34)	8.10 (1.95)
L	73	1.64 (2.37)	4.37 (0.17)	0.83 (1.38)	7.93 (1.27)
40-59					
H	328	25.44 (3.50)	36.96 (0.40)	0.82 (1.57)	8.16 (1.93)
L	373	1.83 (2.47)	4.41 (0.18)	0.81 (1.21)	7.98 (1.33)
60-74					
H	99	23.09 (3.93)	44.08 (0.40)	0.85 (1.36)	9.80 (2.13)
L	97	2.12 (2.66)	4.55 (0.17)	0.79 (1.22)	7.99 (1.33)
≥75					
H	30	27.03 (3.96)	41.12 (0.24)	0.81 (1.50)	7.56 (1.64)
L	46	2.11 (2.24)	4.73 (0.20)	0.82 (1.25)	7.79 (1.29)
Smoking					
Never					
H	436	24.40 (3.58)	37.72 (0.40)	0.80 (1.40)	8.15 (1.92)
L	392	1.43 (2.11)	4.15 (0.18)	0.82 (1.22)	7.89 (1.28)
Former					
H	29	28.46 (2.31)	45.92 (0.29)	0.87(1.36)	10.19 (1.97)
L	43	1.76 (2.52)	4.50 (0.19)	0.86 (1.32)	7.22 (1.58)
Current					
H	98	26.27 (3.63)	42.56 (0.40)	0.85 (1.88)	9.00 (2.11)
L	154	3.79 (2.46)	5.31 (0.17)	0.77 (1.24)	8.34 (1.33)
Alcohol					
Never					
H	364	23.55 (3.57)	39.71 (0.39)	0.82 (1.52)	8.27 (1.99)
L	332	1.77 (2.37)	4.24 (0.18)	0.82 (1.24)	7.95 (1.29)
Former					
H	44	31.05 (2.90)	45.44 (0.28)	0.84 (1.33)	8.36(1.70)
L	46	1.91 (2.55)	4.24 (0.15)	0.86 (1.32)	7.16 (1.56)
Current					
H	155	26.70 (3.58)	35.50 (0.43)	0.81 (1.47)	8.67 (1.97)
L	211	2.03 (2.63)	4.86 (0.17)	0.78 (1.20)	8.15 (1.30)

^a ^*P *< 0.001

### Cancer mortality and rate ratio

Of the 10,688,263 residents of the RAGDP evaluated in the third retrospective investigation, 71,912 deaths, including 14,635 cancer deaths, were recorded from 2004 to 2005. Amongst the 761,224 residents of Wengyuan County, 4,904 deaths, including 1,080 cancer deaths, were recorded from 2004 to 2005. Of the 194,131 residents of the nine villages analyzed in our current study, 1,093 deaths, including 342 cancer deaths, were recorded from 2000-2007. The age-adjusted cancer mortality rates and the expected number of cancer deaths for both sexes, males and females were respectively calculated in each studied population (See Additional file 6, 7, 8 and 9; tables s2, s3, s4 and s5).

The rates of mortality from all types of cancer in the HEA were substantially elevated in comparison with the mortality rate in the LEA for both sexes [rate ratio (RR) = 2.32; 95% confidence interval = 1.87 - 2.89), men (2.13; 1.63 - 2.77) and women (2.83; 1.91 - 4.19), compared with the corresponding rates in the whole of Wengyuan County among both sexes (1.92; 1.66 - 2.20), men (1.81; 1.51 - 2.14) and women (1.95; 1.53 - 2.46). The cancer mortality rates in the HEA were also elevated compared with the rates in the RAGDP population among both sexes (2.01; 1.74 - 2.30), men (1.99; 1.65 - 2.35) and women (2.12; 1.66 - 2.67) (Table 3).

**Table 3:** Comparison of the cancer mortality rates (per 100,000) in high-exposure area (HEA) to those in low-exposure area (LEA), Wengyuan county and the rural areas of Guangdong Province (RAGDP)

Population		All cancers			Esophagus cancer			Stomach cancer			Lung cancer		
					
(Years)	Statistic	Both	Men	Women	Both	Men	Women	Both	Men	Women	Both	Men	Women
The HEA	E^a^	194	125	68	30	17	13	60	34	26	36	24	12
(2000-2007)	Rate^b^	265.1	332.5	191.1	41.0	45.2	36.5	82.0	90.4	73.1	49.2	63.9	33.7
Comparison:	E^a^	138	99	39	22	14	8	19	17	2	27	22	5
The LEA (2000-2007)	Rate^b^	5 114.1	156.3	67.7	18.2	22.1	13.9	15.7	26.9	3.5	22.3	34.7	8.7
	Rate ratio^c^	2.32	2.13	2.83	2.25	2.05	2.63	5.28	3.37	21.06	2.20	1.84	3.89
	95% CI^d^	1.87-2.89	1.63-2.77	1.91-4.19	1.30-3.91	1.01-4.15	1.09-6.35	3.12-8.74	1.88-6.03	5.00-88.71	1.34-3.63	1.03-3.28	1.37-11.03
	P^d^	<0.001	<0.001	<0.001	0.003	0.043	0.025	<0.001	<0.001	<0.001	0.001	0.036	0.006
Comparison: Wengyuan County (2004-2005)	Rate^†^	138.3	183.5	98.0	17.0	16.1	17.6	37.0	48.6	26.4	22.2	38.0	10.3
	Rate ratio^c^	1.92	1.81	1.95	2.41	2.81	2.08	2.22	1.86	2.77	2.22	1.68	3.27
	95% CI^e^	1.66-2.20	1.51-2.14	1.53-2.46	1.66-3.40	1.69-4.41	1.16-3.46	1.71-2.83	1.31-2.57	1.85-4.00	1.58-3.04	1.10-2.46	1.77-5.56
	P^e^	<0.001	<0.001	<0.001	<0.001	<0.001	0.017	<0.001	0.001	<0.001	<0.001	0.018	0.001
Comparison: the RAGDP (2004-2005)	Rate^†^	131.8	167.3	90.3	11.8	16.7	7.1	15.0	20.4	9.9	26.6	39.8	14.6
	Rate ratio^c^	2.01	1.99	2.12	3.48	2.71	5.14	5.46	4.43	7.73	1.85	1.61	2.31
	95% CI^e^	1.74-2.30	1.65-2.35	1.66-2.67	2.39-4.90	1.63-4.25	2.86-8.57	4.21-6.99	3.12-6.12	4.91-10.64	1.32-2.53	1.05-2.35	1.25-3.92
	P^e^	<0.001	<0.001	<0.001	<0.001	<0.001	<0.001	<0.001	<0.001	<0.001	0.001	0.030	0.010

^a^Expected deaths; round to nearest whole number for statistical calculations based on the binomial distribution.

^b ^Calculated by the expected deaths in study regions by the total populations of men or women or both in **Additional file **6**table s2**.

^c ^Calculated by dividing the rate in the exposed regions by the rate in the comparison populations.

^d ^Exact mid-*P *95% CIs and two-side hypothesis test probabilities (*P*) were calculated with PEPI Compare2 program.

^e ^The exact CI and *P *were calculated using PEPI Describe for the Poisson statistical distribution.

Not only all-cancer mortality rate, but also the mortality rates from esophageal-, stomach- and lung-cancer in the HEA were significantly elevated in comparison with the corresponding rates in the LEA, Wengyuan County and the RAGDP population for both sexes, men and women (Table 3).

The mortality rates from leukocythemia and non-Hodgkin's lymphoma in the HEA were strongly elevated in comparison with these rates in the LEA, Wengyuan County and the RAGDP population, but among men only. In contrast, the mortality rates from liver cancer, kidney cancer and other cancers were not elevated in the HEA compared with the corresponding rates in the LEA, Wengyuan County, or the RAGDP population (Table 4).

**Table 4:** Comparison of the cancer mortality rates (per 100,000) in high-exposure area (HEA) to those in low-exposure area (LEA), Wengyuan county and the rural areas of Guangdong Province (RAGDP)

Population		Liver cancer			Leukocythemia			Non-Hodgin disease			**Other cancers**^**f**^		
					
(Years)	Statistic	Both	Men	Women	Both	Men	Women	Both	Men	Women	Both	Men	Women
The HEA	E^a^	38	27	11	8	7	1	4	3	1	21	14	7
(2000-2007)	Rate^b^	51.9	71.8	30.9	10.9	18.6	2.8	5.5	8.0	2.8	28.7	37.2	19.7
Comparison:	E^a^	39	28	11	7	4	4	0	0	0	23	13	10
The LEA	Rate^b^	32.2	44.3	19.1	5.8	6.3	6.9	0	0	0	19.0	20.5	17.4
(2000-2007)	Rate ratio^c^	1.61	1.62	1.62	1.89	2.95	0.41	-	-	-	1.51	1.81	1.13
	95% CI^d^	1.03-2.52	0.96-2.75	0.70-3.74	0.69-5.21	0.86-10.1	0.05-3.62	-	-	-	0.84-2.73	0.85-3.86	0.43-2.98
	P^d^	0.035	0.070	0.254	0.211	0.070	0.403	-	-	-	0.170	0.117	0.799
Comparison: Wengyuan County (2004-2005)	Rate^†^	42.3	64.0	21.5	4.5	5.2	3.8	1.2	1.3	1.1	16.1	10.3	17.3
	Rate ratio^c^	1.23	1.12	1.44	2.43	3.58	0.74	4.58	6.15	2.55	1.78	3.61	1.14
	95% CI^e^	0.88-1.67	0.75-1.61	0.76-2.50	1.13-4.62	1.57-7.10	0.04-3.65	1.44-11.0	1.56-16.7	0.13-12.64	1.11-2.63	1.99-5.73	0.51-2.31
	P^e^	0.209	0.526	0.239	0.026	0.005	0.868	0.002	0.015	0.382	0.018	<0.001	0.650
Comparison: the RAGDP (2004-2005)	Rate^†^	39.8	63.1	16.7	4.0	4.2	3.8	1.8	2.3	1.3	32.8	20.8	36.9
	Rate ratio^c^	1.30	1.14	1.85	2.73	4.41	0.74	3.06	3.48	2.15	0.88	1.79	0.53
	95% CI^e^	0.94-1.77	0.77-1.63	0.97-3.22	1.27-5.18	1.94-8.76	0.04-3.65	0.95-7.20	0.89-9.49	0.11-10.72	0.56-1.31	1.00-2.87	0.24-1.07
	P^e^	0.110	0.483	0.059	0.014	0.001	0.868	0.059	0.068	0.447	0.557	0.051	0.080

^a ^Expected deaths; round to nearest whole number for statistical calculations based on the binomial distribution.

^b ^Calculated by the expected deaths in study regions by the total populations of men or women or both in **Additional file **6**table s2**.

^c ^Calculated by dividing the rate in the exposed regions by the rate in the comparison populations.

^d ^Exact mid-*P *95% CIs and two-side hypothesis test probabilities (*P*) were calculated with PEPI Compare2 program.

^e ^The exact CI and *P *were calculated using PEPI Describe for the Poisson statistical distribution.

^f ^Excluding the cases of leukocythemia and non-Hodgin disease.

### Relationships between heavy metal exposure and the risk of cancer mortality

We initially found by using simple linear regression that exposures to Cd and Pb were associated, respectively, with increased risk of all types of cancer and stomach cancer among both the sexes combined and women, but that Zn exposure was not associated with the risk of site-specific cancer mortality (Table 5 and 6). Interestingly, further multiple linear regression analysis revealed that there was a negative correlation between exposure to Pb and the risk for all cancer mortality, and that no relationships existed between exposure to Cd and Pb and the risk of stomach cancer mortality among both the sexes combined and women, which was counterintuitive. Because there were strong correlations found between the blood Cd and Pb levels (r_men_, _Cd-Pb _= 0.927, and r_women_, _Cd-Pb _= 0.886) in the nine villages evaluated, collinearity needed to be considered as this can skew the observed relationships. We thus employed ridge regressions to analysis these relationships, which revealed that the blood Cd and Pb level exploratory variables have effects upon the age-adjusted mortality rates from all-cancer and stomach cancer among both the sexes combined and women, respectively.

**Table 5:** Estimation of the age-adjusted mortality risk for the selected specific cause associated with blood heavy metals levels for both sexes using simple, multiple linear and ridge regression in the nine villages evaluated in this study

	**Both sexes**^**a**^									
	
	All cancer			Esophageal cancer			Stomach cancer		Lung cancer	
	**B**^**b**^	P		**B**^**b**^	P		**B**^**b**^	P	**B**^**b**^	P
Cd	0.301	0.002^c^		0.082	0.770		0.584	0.012^c^	0.279	0.062
Pb	0.260	0.039^c^		-0.045	0.881		0.559	0.046^c^	0.263	0.117
Zn	-0.119	0.900		0.134	0.944		0.102	0.963	0.107	0.929

	**Both sexes **^**d**^									
		
	**All cancers**					**Stomach cancer**				

	**B**^**c**^	**P**	**Tolerance**^**e**^	**VIF**^**f**^	**RRC**^**j**^	**B**^**c**^	**P**	**Tolerance**^**e**^	**VIF**^**f**^	**RRC**^**h**^

Cd	0.483	0.018^c^	0.172	5.826	0.376616	0.723	0.156	0.168	5.964	0.303854
Pb	-0.216	0.228	0.172	5.826	0.188513	-0.168	0.739	0.168	5.964	0.440660

^a ^using simple linear regression.

^b ^Estimate of the regression coefficient of log-transformed for blood cadmium, lead and zinc levels by using simple linear regression.

^c ^*P *< 0.05.

^d ^Estimate of the regression coefficient of the relationships between log-transformed for blood cadmium and lead levels and log-transformed for age-adjusted mortality rates from all-cancer and stomach cancer using multiple linear regression and estimate of the regression coefficient of the relationships log-transformed for blood cadmium, lead and zinc levels and the original age-adjusted mortality rates from all-cancer and stomach cancer using ridge regression.

^e ^A tolerance <0.10 indicates that collinearity must be considered.

^f ^Variance inflation factor; If >10, collinearity must be considered.

^j ^Ridge regression coefficients; a Ridge *k *of 0.85 and RSQ of 0.60815 were caiculated using ridge regression.

^h ^Ridge regression coefficients; a Ridge *k *of 0.40 and RSQ of 0.66049 were caiculated using ridge regression.

## Discussion

We have conducted a large, death certificate-based ecological epidemiological study using cause-specific cancer mortality ratios, and observed that long-term environmental exposure to Cd and Pb results, at least partly, in a substantial elevation in the risk of mortality from all cancers, in addition to, stomach, esophageal and lung cancer. Our data show that the exposure levels to metal contaminants were high in the HEA with the mean blood Cd concentration (44.5 nmol/L) found to be nearly 13-fold and 5-fold higher, respectively, than that in the LEA and the maximum recommended by the Occupational Safety and Health Administration (OSHA) Safety Standard (5.0 μg/L) [37]. Moreover, the mean blood Cd concentration of the participants living in the HEA was nearly 2.5-fold higher than that previously measured in workers with a 20-year cumulative cadmium exposure and showing a blood cadmium concentration of 10 μg/L [38]. The mean blood Pb level of the subjects in the HEA was found in previous occupational studies to be in the range of 26 μg/dL to 80 μg/dL [11,39]. The duration of exposure was about 30 years among individuals who suffered cancer-related deaths in the HEA in our present study, with a median age of death from stomach cancer for males and females of 73 and 66 years, respectively.

Although the mean blood Zn concentration of the participants living in the HEA was significantly higher than that found in the LEA, further analysis indicated that there was no association between Zn exposure and site-specific cancer mortality. This result is consistent with a previous study showing no evidence for an association between Zn exposure and an increased human cancer risk, particularly for lung or stomach cancer [10].

There is now strong evidence for a positive association between occupational Cd exposure alone and lung cancer risk, and a recent cohort study in an area of low-level Cd pollution in Belgium has reported a significantly positive association between urinary cadmium (U-Cd) levels and the incidence of all cancer and lung cancer in the local population [40]. However, a few population-based studies in Japan have reported inconsistent results in this regard [41-43]. Two further studies on the general population in the United States have shown that U-Cd levels of 0.28 μg/g creatinine in men and blood Pb levels of 5-9 μg/dL could, respectively, significantly increase the risk of death from all cancer [44,45]. However, whether lead causes cancer has not been definitively established [8,39]. The site-specific cancers reported to be associated with lead exposure vary among epidemiological studies. Two previous meta-analysis studies [11,39] have indicated that occupational Pb exposure is associated with an increased risk of all cancer, lung cancer and stomach cancer, but little evidence has been found for an increased risk of kidney or brain cancer from Pb.

Similar to previous analyses, our present findings indicate that the rate of mortality from all types of cancer in the HEA among both sexes, men and women were substantially elevated in comparison with those of the LEA, the whole of Wengyuan County and the RAGDP population. At the population level, our analyses showed not only that Cd exposure but also that Pb exposure was significantly positively correlated with all-cancer mortality among both the sexes combined and women. Moreover, Cd exposure showed a borderline statistically significant correlation with all-cancer mortality among men (*P *= 0.062; Table 6). However, the significantly elevated all-cancer mortality rate could not be distinguished from independent effects or joint action resulting from Cd and Pb.

**Table 6:** Estimation of the age-adjusted mortality risk for the selected specific cause associated with blood heavy metals levels for women and men separately using simple, multiple linear and ridge regression in the nine villages evaluated in this study

	**Women**^**a**^								**Men**^**a**^							
		
	All cancer		Esophageal cancer		Stomach cancer		Lung cancer		All cancers		Esophageal cancer		Stomach cancer		Lung cancer	
	**B**^**b**^	P	**B**^**b**^	P	**B**^**b**^	P	**B**^**b**^	P	**B**^**b**^	P	**B**^**b**^	P	**B**^**b**^	P	**B**^**b**^	P
Cd	0.365	0.001^c^	0.204	0.286	0.549	0.037^c^	0.324	0.067	0.276	0.062	0.472	0.071	0.558	0.070	0.163	0.493
Pb	0.374	0.026^c^	0.175	0.476	0.684	0.020^c^	0.275	0.272	0.221	0.101	0.411	0.624	0.424	0.151	0.194	0.350
Zn	-0.034	0.977	1.361	0.323	0.943	0.691	0.444	0.759	-0.366	0.718	0.235	0.893	-0.280	0.898	-0.312	0.831

	**Women **^**d**^															
		
	**All cancers**								**Stomach cancer**							
	
	**B**^**c**^	**P**		**Tolerance**^**e**^		**VIF**^**f**^	**RRC**^**j**^		**B**^**c**^	**P**		**Tolerance**^**e**^		**VIF**^**f**^	**RRC**^**h**^	

Cd	0.491	0.024^c^		0.197		5.073	0.469722		0.221	0.491		0.245		4.075	0.454158	
Pb	-0.175	0.424		0.197		5.073	0.205384		0.453	0.291		0.245		4.075	0.342160	

^a ^using simple linear regression.

^b ^Estimate of the regression coefficient of log-transformed for blood cadmium, lead and zinc levels by using simple linear regression.

^c ^*P *< 0.05.

^d ^Estimate of the regression coefficient of the relationships between log-transformed for blood cadmium and lead levels and log-transformed for age-adjusted mortality rates from all-cancer and stomach cancer using multiple linear regression and estimate of the regression coefficient of the relationships between log-transformed for blood cadmium, lead and zinc levels and the original age-adjusted mortality rates from all-cancer and stomach cancer using ridge regression.

^e ^A tolerance <0.10 indicates that collinearity must be considered.

^f ^Variance inflation factor; If >10, collinearity must be considered.

^j ^Ridge regression coefficients; a Ridge *k *of 0.50 and RSQ of 0.69853 were calculated using ridge regression.

^h ^Ridge regression coefficients; a Ridge *k *of 0.10 and RSQ of 0.66647 were calculated using ridge regression.

In terms of specific cancers, the rates of mortality from stomach cancer in the HEA among both sexes, men and women was substantially elevated in comparison with the corresponding rates the LEA, and the Wengyuan county and RAGDP populations. Only one previous cohort study [46] has reported a statistically significant positive association between occupational Cd exposure and stomach cancer mortality (SMR = 139; 95% CI, 111-166). Previous meta-analyses have reported that a high occupational Pb exposure is associated with an increased risk of stomach cancer [risk ratio (RR) = 1.33, 95% CI, 1.18-1.49 and RR = 1.34, 95% CI, 1.14-1.57, respectively]. To date however, there has been no substantial evidence of the association between dietary Cd or Pb intake and risk of stomach cancer mortality in the general population [15,45]. This could be attributed to different exposure pathways, exposure doses and threshold effects. A study by Jemal et al (2002) has reported a threshold effect in women, with the cancer risk becoming significantly elevated at a blood Pb concentration of approximately 24 μg/dL [47].

Potential risk factors for stomach cancer in China include *Helicobacter Pylori *(H. Pylori) infection of the stomach, a family history of stomach cancer, smoking, alcohol, consumption of salted foods, and a low intake of green vegetables and fruit, vitamin C, and calcium [48]. Although individual-level confounding factors could not be controlled for in the present study, such as *Helicobacter Pylori *infection of the stomach, smoking or excessive drinking, the populations in the HEA and LEA showed similar SES, dietary and geographic characteristics, which may limit any potential biases. In particular, women generally did not have smoking or drinking habits in the Hengshihe River regions. In addition, the two reference populations were representative of the average and age-adjusted stomach cancer mortality rates resulting from all potential risk factors. Although there might be other potential risk factors, further analysis at the population level showed that Cd and Pb exposures were associated, respectively, with a significantly increased rate of stomach cancer among both the sexes combined and women and that Cd exposure had a borderline statistically significant correlation with all-cancer mortality among men (*P *= 0.070, Table 6). Hence, our findings revealed a strong association between stomach cancer risk and environmental exposure to both Cd and Pb, but additional follow up studies focusing on both heavy metals are necessary to confirm these relationships.

To date, there has been no strong evidence for an association between dietary Cd or Pb intake and the risk of esophageal cancer mortality in the general population [15,45]. One study has reported that the incidence of malignant tumors in Nanao island in the South China sea has markedly increased during the period 1995-2003, particularly esophageal cancer (71.07/10^5^) and cardiac cancer (34.59/10^5^) [49]. Element analysis has also shown that the levels of Pb and Cd in the hair in a Nanao high-risk population were higher than those in a Meizhou Hakka control population, which may indicate a possible association with esophageal cancer risk. In terms of the associations between Cd and Pb exposure and esophageal cancer mortality in the HEA in our present report, the low pH of source water samples and water from some wells may be potential confounding factors. The low pH values for the water and soil also increase Cd and Pb bioavailability. The softer waters in the HEA are acidic in character and could well contain a number of toxic substances greatly in excess of the levels presented, such as Cd and Pb. In addition, soft water is more corrosive than hard water and promotes the dissolution of Cd and Pb from contaminated water sources [50]. Hence, the presence of soft water in combination with exposure to both Cd and Pb might further increase the risk of carcinogenicity and play a role in esophageal cancer etiology. A 42% excess risk of mortality from esophageal cancer in relation to the use of soft water (adjusted odds ratio and 95% confidence intervals; 1.42 (1.22-1.66)) has been reported in a study on Taiwan's drinking water [51]. Further studies will be required however to validate these phenomena. It is noteworthy also that some potential risk factors related to esophageal cancer, such as smoking, drinking, a low SES, poor nutrition, family history, human papillomavirus (HPV) infection and p53 mutations [52] could not be controlled for in our present study. At the population level, however, our analyses show that Cd exposure has a borderline statistically significant correlation with esophageal cancer mortality among men (*P *= 0.071, Table 6). Therefore, our findings suggest a possible association between exposure to Cd and Pb and the risk of esophageal cancer mortality in a local environment. Due to its high occurrence in the HEA, esophageal cancer mortality should be further investigated using a cohort study.

With respect to lung cancer, and similar to previous analysis, our current study demonstrates that a long-term environmental exposure to both Cd and Pb is associated with an elevated risk of lung cancer. The main known risk factors for lung cancer are cigarette smoking, occupational exposure to toxic substances, environmental pollution, genetic polymorphisms, a family history of lung cancer and other tumor and respiratory system diseases [53]. Although these risk factors could not be controlled for in our present analysis, other potential risk factors were equivalent in the HEA and LEA populations, such as language, a similar SES and geographic characteristics, little occupational exposures and low air pollution. Moreover, at the population level, our analyses show that Cd exposure has a borderline statistically significant correlation with lung cancer mortality among the sexes combined (*P *= 0.062, Table 5) and women (*P *= 0.067, Table 6). Hence, our data reporting a strong association between long-term environmental exposure to Cd and Pb and lung cancer risk are valid, but further cohort studies are necessary to make a definitive conclusion.

In our present investigation, a significant mortality increase from leukocythemia and non-Hodgkin's lymphoma was found among men only (Table 4). Seven deaths occurred from leukocythemia (ICD-10; C91-95) and three from non-Hodgkin's lymphoma (ICD-10; C82-85) in the HEA for men. Only one previous study has shown that occupational Cd exposure is linked to tumors of the hematopoietic system [54]. Oral exposure to Cd has also been reported to induce tumors of the hematopoietic system in rats and mice [55]. Thus, the available data seem to suggest a potential association between the exposure to Cd and Pb and tumors of the hematopoietic system among men only. However, it should be noted that number of deaths among men in our current cohorts was small, and potential confounding factors in some of these deaths could not be ruled out. One of the known risk factors for tumors of the hematopoietic system is Epstein-Barr virus infection [56,57]. Moreover, the development of leukemia is a complex process involving many risk factors, such as diagnostic X-rays, the history of occupational exposure of organic solvents (2.56, 1.03-6.39), the moving interval after house decoration (1.67, 1.173-2.39), the years of oxidant hair dye use (1.68, 1.04-2.70), and smoking and pesticide application [58,59]. Such information was not available in the present study and further cohort studies are necessary to confirm these relationships.

No increase in mortality from liver, kidney, bladder, brain or prostate cancer was found in the present study. One meta-analysis report has commented that increases in kidney and bladder cancer reported in the literature could have been the result of publication bias. The only way to resolve this issue is to conduct a comprehensive meta-analysis based on the data available from all relevant studies (published or otherwise).

Several limitations of our present study need to be mentioned. First, there may be an ecological fallacy, which means the same relationship exists among all of the individuals tested [60]. An individual-level confounding factor is particularly difficult to control and may have affected the results. However, an ecological study is especially useful when individual data are unavailable or are unlikely to be accurately reported by individual respondents because of an older age or lack of education. Second, there was a lack of more detailed cancer risk factor information for the studied population. Third, a larger number of observed cancer deaths will still be required to validate our results. Fourth, single blood measurements of multiple heavy metals, which are imperfect biomarkers of chronic exposure, were used in our analyses. Environmental exposures, however, are likely to be less changeable than occupational exposures, particularly for long-term local residents of the nine studied villages. Moreover, population studies are frequently based on single blood levels [45,61,62]. Fifth, our present examination of the correlations between exposure to multiple heavy metals and the risk of site-specific cancer mortality in the nine studied villages provides only indirect evidence of the potential risk factors related to cancer. The blood metal concentrations may be variable and may not be representative of the population dying of cancer during observation period.

Despite these limitations however, our current study does address the public concern about whether the detrimental effects of heavy metal pollution are higher in the areas around the Dabaoshan mine than in other rural areas. Many sources of drinking and irrigation water have been contaminated by multiple heavy metals, such as Cd and Pb, with advent of industrialization especially in developing countries like China [63]. Such contaminations have become a serious health hazard for millions of people. In addition, a cross-sectional study showed that some residents noted the risk of heavy-metal pollution and did not consume produce grown on the local farms in the HEA. Based on our findings therefore, effective and preventive measures must be immediately taken in some of the polluted areas, such as improvements to the production process to better control pollution sources, to ban the drinking and use of contaminated water, the building of a new water reservoir and new management of soil and planting economic crops replacing agriculture crops.

The strengths of our current study stem from the death certificate-based retrospective mortality approach and the use of a population with a documented long-term environmental exposure to heavy metals. Furthermore, blood Cd and Pb concentrations are biomarkers of internal doses, which integrate all routes of exposure. Following long-term exposure to low levels of environmental Cd, the blood Cd concentration may be a good indicator of the total Cd body burden [64].

## Conclusions

Our current findings reveal a strong association between long-term environmental exposure to Cd and Pb and an increased risk of esophageal-cancer, stomach- and lung-, and all cancer mortality, although our ecological mortality study design does not allow for firm conclusions to be drawn at this time. Although further research is required to explore the effects of multiple heavy metals on cancer risk based on cohort studies, our study provides useful new insights into the causes of several types of cancer mortality and the possibility of reducing these risks through environmental interventions.

## Competing interests

The authors declare that they have no competing interests.

## Authors' contributions

MW and WHL contributed to the study design, data analysis, and writing. WHL, HS and YY participated in the study design and administered the study. W-QC, CYL, QSH, and ZFR were responsible for the field investigations and data collection. YJX, and AMZ organized the field investigations. All authors read and approved the final manuscript.

## Pre-publication history

The pre-publication history for this paper can be accessed here:

http://www.biomedcentral.com/1471-2458/11/319/prepub

## Supplementary Material

Additional file 1 **The number of blood Cd levels (μg/L) in the residents living in the high-exposure area**. The figure provided the number of blood Cd levels in the residents living in the high-exposure area of this study. Click here for file

Additional file 2 **The number of blood Pd levels (μg/dL) in the residents living in the high-exposure area**. The figure provided the number of blood Pd levels in the residents living in the high-exposure area of this study. Click here for file

Additional file 3 **The number of blood Cd levels (μg/L) in the residents living in the low-exposure area**. The figure provided the number of blood Cd levels in the residents living in the low-exposure area of this study. Click here for file

Additional file 4 **The number of blood Pd levels (μg/dL) in the residents living in the low-exposure area**. The figure provided the number of blood Pd levels in the residents living in the low-exposure area of this study. Click here for file

Additional file 5 **Blood levels of cadmium, lead, copper and zinc in study subpopulations presented by participant characteristics**. The table described the blood levels of cadmium, lead, copper and zinc in study subpopulations by participant characteristics, including sex and each village. Click here for file

Additional file 6 **The study populations in the mortality observation period were supplied from the Centre for Disease Control and Prevention of Wengyuan County in 2008**. The table showed the number of the study populations in the mortality observation period of this study. Click here for file

Additional file 7 **Mortality data for all subjects from the study regions near the Dabaoshan mine for which the cancer rates (per 100,000) for 2000-2007 as calculated in the present study**. The table showed the mortality data for all subjects, including observed deaths, crude rate, age-adjusted rate and expected deaths, from the study regions near the Dabaoshan mine for which the cancer rates for 2000-2007 as calculated of this study. Click here for file

Additional file 8 **Mortality data for men from the study regions near the Dabaoshan mine for which the cancer rates (per 100,000) for 2000-2007 as calculated in the present study**. The table showed the mortality data for men, including observed deaths, crude rate, age-adjusted rate and expected deaths, from the study regions near the Dabaoshan mine for which the cancer rates for 2000-2007 as calculated of this study. Click here for file

Additional file 9 **Mortality data for women from the study regions near the Dabaoshan mine for which the cancer rates (per 100,000) for 2000-2007 as calculated in the present study**. The table showed the mortality data for women, including observed deaths, crude rate, age-adjusted rate and expected deaths, from the study regions near the Dabaoshan mine for which the cancer rates for 2000-2007 as calculated of this study. Click here for file
