# Supplementary figures and images for: Cancer mortality in a Chinese population surrounding a multi-metal sulphide mine in Guangdong province: an ecologic study
Source: BMC Public Health. 2011 May 16;11:319. doi: 10.1186/1471-2458-11-319 (PMC3112132; doi:10.1186/1471-2458-11-319)

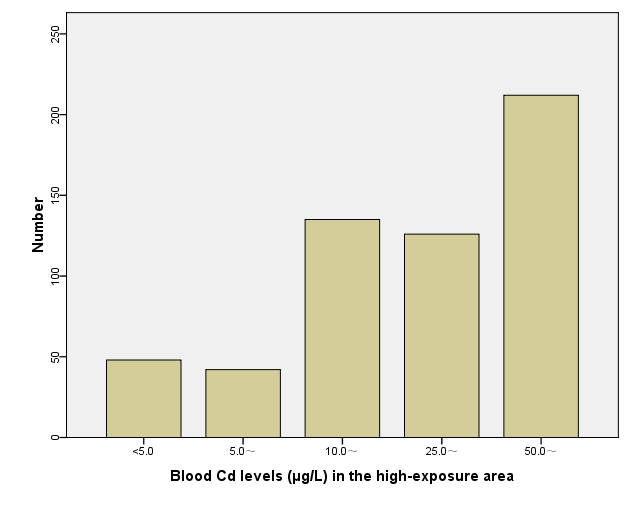


Figure s1 -The number of blood Cd levels (μg/L) in the residents living in the high-exposure area

Supplement: Additional file 1 — The number of blood Cd levels (μg/L) in the residents living in the high-exposure area. The figure provided the number of blood Cd levels in the residents living in the high-exposure area of this study. [file 1471-2458-11-319-S1.DOC]

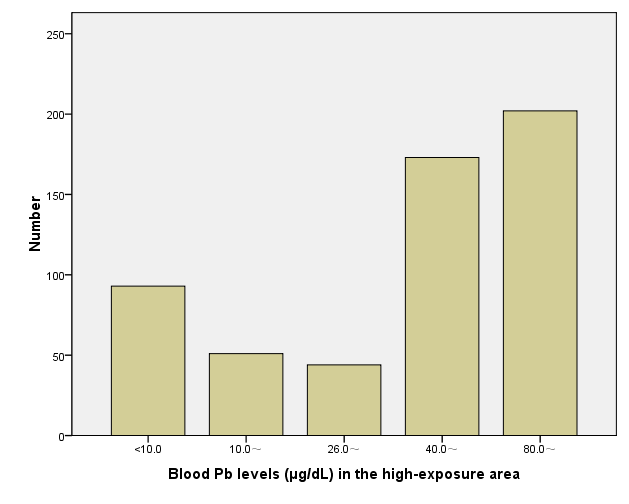


Figure s2- The number of blood Pd levels (μg/dL) in the residents living in the high-exposure area.

Supplement: Additional file 2 — The number of blood Pd levels (μg/dL) in the residents living in the high-exposure area. The figure provided the number of blood Pd levels in the residents living in the high-exposure area of this study. [file 1471-2458-11-319-S2.DOC]

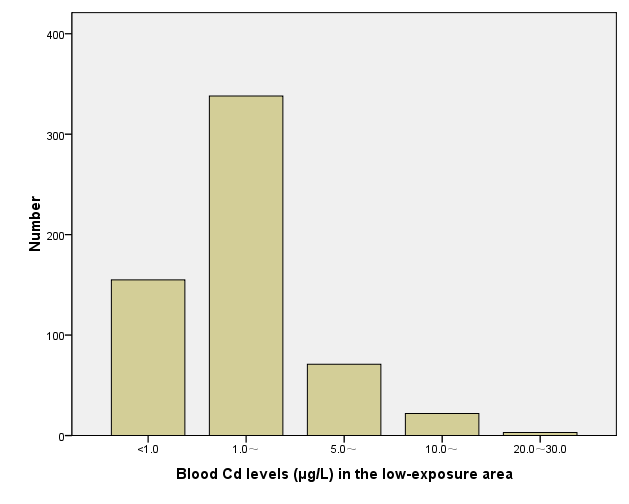


Figure s3 - The number of blood Cd levels (μg/L) in the residents living in the low-exposure area.

Supplement: Additional file 3 — The number of blood Cd levels (μg/L) in the residents living in the low-exposure area. The figure provided the number of blood Cd levels in the residents living in the low-exposure area of this study. [file 1471-2458-11-319-S3.DOC]

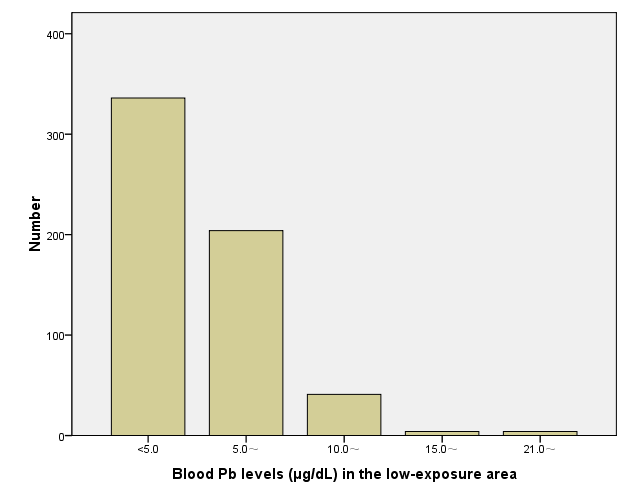


Figure s4 - The number of blood Pd levels (μg/dL) in the residents living in the low-exposure area.

Supplement: Additional file 4 — The number of blood Pd levels (μg/dL) in the residents living in the low-exposure area. The figure provided the number of blood Pd levels in the residents living in the low-exposure area of this study. [file 1471-2458-11-319-S4.DOC]
