# Supplementary material for: Cancer mortality in a Chinese population surrounding a multi-metal sulphide mine in Guangdong province: an ecologic study
Source: BMC Public Health. 2011 May 16;11:319. doi: 10.1186/1471-2458-11-319 (PMC3112132; doi:10.1186/1471-2458-11-319)
Supplement: Additional file 5 — Blood levels of cadmium, lead, copper and zinc in study subpopulations presented by participant characteristics. The table described the blood levels of cadmium, lead, copper and zinc in study subpopulations by participant characteristics, including sex and each village. [file 1471-2458-11-319-S5.DOC]

**Table s1** -Blood levels of cadmium, lead, copper and zinc in study subpopulations presented by participant characteristics

|  | Geometric mean (SD) | | | | |
| --- | --- | --- | --- | --- | --- |
| Characteristic | n | Cadmium (µg/L) | Lead (µg/dL) | Copper (mg/L) | Zinc (mg/L) |
| High-exposure area (І-Ⅲ) | 563 | 24.10 (3.52)* | 38.91 (0.39)* | 0.82 (1.49) | 8.38 (1.96)* |
| Shangba (І)  Men  Women  Xiaozhen (Ⅱ)  Men  Women  Dongfang (Ⅲ)  Men  Women | 198  88  110  177  77  100  188  75  113 | 34.80 (2.96)  35.76 (2.65)  34.05 (3.21)  20.17 (4.38)  10.33 (4.16)  33.77 (3.68)  21.37 (3.11)  21.37 (2.96)  21.37 (3.22) | 67.36 (0.24)  67.73 (0.24)  67.08 (0.25)  12.72 (0.43)  10.35 (0.40)  14.91 (0.45)  62.55 (0.28)  74.15 (0.27)  55.87 (0.29) | 0.84 (1.42)  0.76 (1.36)  0.90 (1.45)  0.75 (1.27)  0.73 (1.26)  0.76 (1.27)  0.86 (1.71)  0.88 (2.05)  0.85 (1.45) | 11.23 (1.75)  10.14 (1.76)  12.19 (1.72)  5.65 (1.68)  5.52 (1.45)  5.75 (1.84)  8.94 (2.09)  9.85(2.23)  8.38 (1.99) |
| Low-exposure area (Ⅳ-Ⅸ) | 589 | 1.87 (2.48) * | 4.46 (0.18) * | 0.81 (1.24) | 7.96 (1.32)* |
| Zhongxin (Ⅳ)  Men  Women | 172  80  92 | 1.70 (2.34)  2.48 (252)  1.22 (1.86) | 5.81 (0.17)  6.19 (0.19)  5.49 (0.16) | 0.86 (1.27)  0.82 (1.19)  0.91 (1.31) | 8.47 (1.34)  8.63 (1.37)  8.34 (1.31) |
| Shaping (Ⅴ)  Men  Women | 72  32  40 | 2.06 (2.57)  2.65 (2.76)  1.68 (2.32) | 4.70 (0.18)  5.22 (0.19)  4.31 (0.17) | 0.81 (1.16)  0.78 (1.20)  0.83 (1.12) | 7.80 (1.30)  8.52 (1.31)  7.27 (1.28) |
| Shuikou (Ⅵ)  Men  Women | 72  30  42 | 1.90 (2.51)  2.51 (3.22)  1.57 (1.92) | 3.79 (0.15)  4.32 (0.15)  3.45 (0.15) | 0.85 (1.29)  0.83 (1.40)  0.87 (1.20) | 8.34 (1.19)  8.39 (1.19)  8.30 (1.20) |
| Fengshan (Ⅶ)  Men  Women | 104  41  63 | 1.23 (2.45)  1.80 (2.72)  0.96 (2.07) | 4.34 (0.18)  4.50 (0.18)  4.24 (0.19) | 0.79 (1.14)  0.75 (1.13)  0.83 (1.14) | 8.17 (1.24)  8.63 (1.28)  7.88 (1.20) |
| Mashan (Ⅷ)  Men  Women | 86  35  51 | 2.15 (2.12)  2.85 (2.15)  1.78 (1.99) | 2.97 (0.19)  3.00 (0.21)  2.95 (0.18) | 0.72 (1.19)  0.68 (1.16)  0.76 (1.21) | 7.51 (1.27)  7.86 (1.34)  7.29 (1.21) |
| Madun (Ⅸ)  Men  Women | 83  38  45 | 3.03 (2.40)  4.30 (2.35)  2.26 (2.20) | 4.47 (0.15)  4.10 (0.16)  4.82 (0.14) | 0.76 (1.25)  0.71 (1.19)  0.81 (1.27) | 7.01 (1.45)  7.48 (1.28)  6.64 (1.56) |
| Sex |  |  |  |  |  |
| Men |  |  |  |  |  |
| High-exposure area | 263 | 20.56 (3.52) | 38.81 (0.40) | 0.79 (1.59) | 8.43(1.92) |
| Low-exposure area | 251 | 2.66 (2.66) | 4.75 (0.19) | 0.77 (1.23) | 8.35 (1.32) |
| Women |  |  |  |  |  |
| High-exposure area | 300 | 29.48 (3.44) | 39.00 (0.39) | 0.84 (1.40) | 8.34 (2.00) |
| Low-exposure area | 338 | 1.43 (2.10) | 4.27 (0.17) | 0.84 (1.24) | 7.68 (1.32) |
| *P＜0.001 |  |  |  |  |  |
