# Supplementary material for: Cancer mortality in a Chinese population surrounding a multi-metal sulphide mine in Guangdong province: an ecologic study
Source: BMC Public Health. 2011 May 16;11:319. doi: 10.1186/1471-2458-11-319 (PMC3112132; doi:10.1186/1471-2458-11-319)
Supplement: Additional file 6 — The study populations in the mortality observation period were supplied from the Centre for Disease Control and Prevention of Wengyuan County in 2008. The table showed the number of the study populations in the mortality observation period of this study. [file 1471-2458-11-319-S6.DOC]

**Table s2** -The study populations in the mortality observation period were supplied from the Centre for Disease Control and Prevention of Wengyuan County in 2008

|  | Wengyuan | Village (Mortality Study Regions No.) | | | | | | | | |
| --- | --- | --- | --- | --- | --- | --- | --- | --- | --- | --- |
| Year | Countya | Shangba (Ⅰ) | Xiaozhen (Ⅱ) | Dongfang (Ⅲ) | Zhongxin (Ⅳ) | Shaping (Ⅴ) | Shuikou (Ⅵ) | Mashan (Ⅶ) | Fengshan (Ⅷ) | Madun (Ⅸ) |
| 2007 | 390,060 | 3,481 | 2,956 | 2,977 | 4,535 | 1,820 | 1,979 | 2,381 | 2,792 | 2,089 |
| 2006 | 387,512 | 3,423 | 2,942 | 2,959 | 4,535 | 1,828 | 1,980 | 2,383 | 2,794 | 2,085 |
| 2005 | 382,012 | 3,385 | 2,926 | 2,936 | 4,524 | 1,800 | 1,970 | 2,369 | 2,797 | 2,074 |
| 2004 | 379,212 | 3,348 | 2,917 | 2,915 | 4,463 | 1,776 | 1,925 | 2,269 | 2,772 | 2,049 |
| 2003 | 376,992 | 3,335 | 2,896 | 2,866 | 4,410 | 1,746 | 1,882 | 2,164 | 2,765 | 2,022 |
| 2002 | 375,607 | 3,304 | 2,877 | 2,834 | 4,402 | 1,735 | 1,881 | 2,163 | 2,737 | 2,011 |
| 2001 | 372,521 | 3,298 | 2,873 | 2,812 | 4,401 | 1,730 | 1,881 | 2,154 | 2,729 | 2,005 |
| 2000 | 370,550 | 3,267 | 2,864 | 2,783 | 4,581 | 1,744 | 1,883 | 2,168 | 2,751 | 2,023 |
| Total | 3,034,466 | 26,841^b^ | 23,251^b^ | 23,082^b^ | 35,851^c^ | 13,179^c^ | 15,381^c^ | 18,051^c^ | 22,137^c^ | 16,358^c^ |
| Sex  N |  | Men Women 13,788 13,053 | Men Women  11,944 11,307 | Men Women  11,857 11,225 | Men Women  18,815 17,036 | Men Women  7,005 6,174 | Men Women  7,841 7,540 | Men Women  9,278 8,773 | Men Women  11,535 10,602 | Men Women  8,846 7,512 |
| ^a^ In Wengyuan county total persons in mortality observation period (2004-2005) has the population of 761,224 (men 391,041 and women 371,083).  ^b^ In HEA (Ⅰ-Ⅲ) total persons in mortality observation period (2000-2007)) has the population of 73,174 (men 37,589 and women 35,585).  ^c^ In LEA (Ⅳ-Ⅸ) total persons in mortality observation period (2000-2007) has the population of 120,957 (men 63,320 and women 57,637). | | | | | | | | | | |
