# Supplementary material for: Cancer mortality in a Chinese population surrounding a multi-metal sulphide mine in Guangdong province: an ecologic study
Source: BMC Public Health. 2011 May 16;11:319. doi: 10.1186/1471-2458-11-319 (PMC3112132; doi:10.1186/1471-2458-11-319)
Supplement: Additional file 7 — Mortality data for all subjects from the study regions near the Dabaoshan mine for which the cancer rates (per 100,000) for 2000-2007 as calculated in the present study. The table showed the mortality data for all subjects, including observed deaths, crude rate, age-adjusted rate and expected deaths, from the study regions near the Dabaoshan mine for which the cancer rates for 2000-2007 as calculated of this study. [file 1471-2458-11-319-S7.DOC]

**Table s3 -**Mortality data for all subjects from the study regions near the Dabaoshan mine for which

the cancer rates (per 100,000) for 2000-2007 as calculated in the present study

|  | Village (mortality study region number) | | | | | | | | |
| --- | --- | --- | --- | --- | --- | --- | --- | --- | --- |
| Type of data  (ICD-10) | Shangba  (Ⅰ) | Xiaozhen  (Ⅱ) | Dongfang  (Ⅲ) | Zhongxin  (Ⅳ) | Shaping  (Ⅴ) | Shuikou  (Ⅵ) | Fengshan  (Ⅶ) | Mashan  (Ⅷ) | Madun  (Ⅸ) |
| Heavy metal contami-  nation^a^ | Yes | Yes | Yes | No | No | No | No | No | No |
| All cancer (C00-C97) |  |  |  |  |  |  |  |  |  |
| Observed deaths | 70 | 74 | 58 | 27 | 17 | 22 | 29 | 26 | 19 |
| Crude rate^b^ | 260.80 | 318.27 | 251.28 | 75.31 | 128.99 | 143.03 | 131.00 | 144.04 | 116.15 |
| Age-adjusted rate^c^ | 248.47 | 309.62 | 240.69 | 74.10 | 121.40 | 146.19 | 125.68 | 144.93 | 116.80 |
| Expected deaths^d^ | 66.69 | 71.99 | 55.55 | 26.57 | 16.00 | 22.49 | 27.82 | 26.16 | 19.11 |
| Esophagus cancer (C15) |  |  |  |  |  |  |  |  |  |
| Observed deaths | 21 | 8 | 1 | 4 | 0 | 5 | 6 | 3 | 5 |
| Crude rate^b^ | 78.24 | 34.41 | 4.33 | 11.16 | 0 | 32.51 | 27.10 | 16.62 | 30.57 |
| Age-adjusted rate^c^ | 78.07 | 32.99 | 4.03 | 9.63 | 0 | 28.95 | 25.00 | 16.68 | 31.84 |
| Expected deaths^d^ | 20.95 | 7.67 | 0.93 | 3.45 | 0 | 4.45 | 5.53 | 3.01 | 5.21 |
| Stomach cancer (C16) |  |  |  |  |  |  |  |  |  |
| Observed deaths | 19 | 17 | 28 | 3 | 7 | 4 | 2 | 4 | 0 |
| Crude rate^b^ | 70.79 | 73.12 | 121.31 | 8.37 | 53.12 | 26.01 | 9.03 | 22.16 | 0 |
| Age-adjusted rate^c^ | 66.67 | 66.80 | 115.07 | 7.62 | 50.39 | 22.10 | 9.50 | 22.40 | 0 |
| Expected deaths^d^ | 17.89 | 15.53 | 26.56 | 2.73 | 6.64 | 3.40 | 2.10 | 4.04 | 0 |
| Liver cancer (C22) |  |  |  |  |  |  |  |  |  |
| Observed deaths | 10 | 18 | 10 | 6 | 4 | 6 | 7 | 6 | 8 |
| Crude rate^b^ | 37.26 | 77.42 | 43.32 | 16.74 | 30.35 | 39.01 | 31.62 | 33.24 | 48.91 |
| Age-adjusted rate^c^ | 37.03 | 80.17 | 41.86 | 16.55 | 26.91 | 42.61 | 34.14 | 40.44 | 47.60 |
| Expected deaths^d^ | 9.94 | 18.64 | 9.66 | 5.93 | 3.54 | 6.55 | 7.56 | 7.30 | 7.79 |
| Lung cancer (C33-C34) |  |  |  |  |  |  |  |  |  |
| Observed deaths | 11 | 15 | 10 | 8 | 2 | 6 | 6 | 4 | 2 |
| Crude rate^b^ | 40.98 | 64.51 | 43.32 | 22.31 | 15.18 | 39.01 | 27.10 | 22.16 | 12.23 |
| Age-adjusted rate^c^ | 40.13 | 64.16 | 42.96 | 21.23 | 14.18 | 40.29 | 25.54 | 22.40 | 11.34 |
| Expected deaths^d^ | 10.77 | 14.92 | 9.92 | 7.57 | 1.87 | 6.19 | 5.65 | 4.04 | 1.85 |
| Other cancers^e^ |  |  |  |  |  |  |  |  |  |
| Observed deaths | 9 | 16 | 9 | 6 | 4 | 1 | 8 | 9 | 4 |
| Crude rate^b^ | 33.53 | 68.81 | 38.99 | 16.74 | 30.35 | 6.50 | 36.14 | 49.86 | 24.45 |
| Age-adjusted rate^c^ | 33.31 | 66.28 | 38.12 | 16.54 | 28.99 | 8.39 | 32.98 | 46.37 | 22.80 |
| Expected deaths^d^ | 8.94 | 15.41 | 8.80 | 5.93 | 3.82 | 1.29 | 7.30 | 8.37 | 3.73 |
| ^a^ Based on exposure levels monitored in the 9 villages in 2006 (see **Table 1-2**). | | | | | | | | | |
| ^b^ Numbers in these rows were calculated by respectively dividing the observed deaths by the total number of men or women  or both as presented in **Additional file 6 table s2**. | | | | | | | | | |
| ^c^ Adjusted to China’s age distribution in 2000. | | | | | | | | | |
| ^d^ Expected deaths were calculated by multiplying the age-adjusted rate by the total number of populations as presented  in **Additional file 6 table s2**.  ^e^ Including 8 cases of leukocythemia, 4 cases of non-Hodgkin lymphoma, 6 cases of colorectal cancer, 5 cases of  nasopharyngeal carcinoma, 4 cases of bone cancer, 1 case of scalp cancer, 1 case of prostate cancer, 1 case of bladder  cancer, 3 cases of breast cancer and 1 case of cervix cancer in the HEA and 8 cases of leukocythemia, 7 cases of colorectal cancer, 3 cases of breast cancer, 2 cases of brain cancer and 12 cases of nasopharyngeal carcinoma in the LEA. | | | | | | | | | |
