# Supplementary material for: Cancer mortality in a Chinese population surrounding a multi-metal sulphide mine in Guangdong province: an ecologic study
Source: BMC Public Health. 2011 May 16;11:319. doi: 10.1186/1471-2458-11-319 (PMC3112132; doi:10.1186/1471-2458-11-319)
Supplement: Additional file 8 — Mortality data for men from the study regions near the Dabaoshan mine for which the cancer rates (per 100,000) for 2000-2007 as calculated in the present study. The table showed the mortality data for men, including observed deaths, crude rate, age-adjusted rate and expected deaths, from the study regions near the Dabaoshan mine for which the cancer rates for 2000-2007 as calculated of this study. [file 1471-2458-11-319-S8.DOC]

**Table s4 -**Mortality data for men in the study regions near the Dabaoshan mine for which the

cancer rates (per 100,000) for 2000-2007 as calculated in the present study

|  | Village (mortality study region number) | | | | | | | | |
| --- | --- | --- | --- | --- | --- | --- | --- | --- | --- |
| Type of data  (ICD-10) | Shangba  (Ⅰ) | Xiaozhen  (Ⅱ) | Dongfang  (Ⅲ) | Zhongxin  (Ⅳ) | Shaping  (Ⅴ) | Shuikou  (Ⅵ) | Fengshan  (Ⅶ) | Mashan  (Ⅷ) | Madun  (Ⅸ) |
| Heavy metal contami-  nation^a^ | Yes | Yes | Yes | No | No | No | No | No | No |
| All cancer (C00-C97) |  |  |  |  |  |  |  |  |  |
| Observed deaths | 42 | 49 | 38 | 20 | 12 | 18 | 22 | 18 | 10 |
| Crude rate^b^ | 304.61 | 410.25 | 320.49 | 106.30 | 171.31 | 229.56 | 190.72 | 194.01 | 113.05 |
| Age-adjusted rate^c^ | 292.39 | 398.51 | 315.13 | 105.30 | 160.50 | 239.67 | 184.05 | 197.08 | 107.23 |
| Expected deaths^d^ | 40.31 | 47.60 | 37.36 | 19.81 | 11.24 | 18.79 | 21.23 | 18.28 | 9.49 |
| Esophagus cancer (C15) |  |  |  |  |  |  |  |  |  |
| Observed deaths | 12 | 5 | 0 | 2 | 0 | 2 | 5 | 2 | 4 |
| Crude rate^b^ | 87.03 | 41.86 | 0 | 10.63 | 0 | 25.51 | 43.35 | 21.56 | 45.22 |
| Age-adjusted rate^c^ | 85.77 | 38.92 | 0 | 9.87 | 0 | 20.76 | 39.23 | 20.67 | 45.54 |
| Expected deaths^d^ | 11.85 | 4.65 | 0 | 1.86 | 0 | 1.63 | 4.53 | 1.92 | 4.03 |
| Stomach cancer (C16) |  |  |  |  |  |  |  |  |  |
| Observed deaths | 11 | 11 | 14 | 3 | 7 | 4 | 1 | 3 | 0 |
| Crude rate^b^ | 79.78 | 92.10 | 118.07 | 15.95 | 99.93 | 51.01 | 8.61 | 32.34 | 0 |
| Age-adjusted rate^c^ | 75.58 | 84.48 | 114.54 | 14.48 | 94.73 | 43.36 | 8.05 | 33.29 | 0 |
| Expected deaths^d^ | 10.42 | 10.09 | 13.58 | 2.72 | 6.64 | 3.40 | 0.93 | 3.08 | 0 |
| Liver cancer (C22) |  |  |  |  |  |  |  |  |  |
| Observed deaths | 7 | 13 | 7 | 5 | 1 | 5 | 6 | 5 | 4 |
| Crude rate^b^ | 50.77 | 108.84 | 59.04 | 26.58 | 14.28 | 63.77 | 52.02 | 53.89 | 45.22 |
| Age-adjusted rate^c^ | 49.40 | 112.90 | 59.00 | 26.90 | 13.30 | 72.48 | 57.30 | 66.10 | 41.65 |
| Expected deaths^d^ | 6.81 | 13.48 | 7.00 | 5.06 | 0.93 | 5.68 | 6.60 | 6.13 | 3.68 |
| Lung cancer (C33-C34) |  |  |  |  |  |  |  |  |  |
| Observed deaths | 5 | 10 | 9 | 6 | 2 | 6 | 5 | 3 | 1 |
| Crude rate^b^ | 36.26 | 83.72 | 75.91 | 31.89 | 28.55 | 76.52 | 43.55 | 32.34 | 11.31 |
| Age-adjusted rate^c^ | 35.53 | 83.35 | 75.42 | 30.18 | 26.51 | 78.95 | 40.85 | 33.29 | 10.23 |
| Expected deaths^d^ | 4.90 | 9.96 | 8.94 | 5.67 | 1.86 | 6.19 | 4.71 | 3.08 | 0.90 |
| Other cancers^e^ |  |  |  |  |  |  |  |  |  |
| Observed deaths | 7 | 10 | 8 | 4 | 2 | 1 | 5 | 5 | 1 |
| Crude rate^b^ | 50.77 | 83.72 | 67.47 | 21.26 | 28.55 | 12.75 | 43.35 | 53.89 | 11.31 |
| Age-adjusted rate^c^ | 51.01 | 79.46 | 66.17 | 20.75 | 27.12 | 16.48 | 38.67 | 48.72 | 9.81 |
| Expected deaths^d^ | 7.03 | 9.49 | 7.85 | 3.90 | 1.90 | 1.29 | 4.46 | 4.52 | 0.87 |
| ^a^ Based on exposure levels monitored in the 9 villages in 2006 (see **Table 1-2**). | | | | | | | | | |
| ^b^ Numbers in these rows were calculated by respectively dividing the observed deaths by the total number of men or women  or both as presented in **Additional file 6 table s2**. | | | | | | | | | |
| ^c^ Adjusted to China’s age distribution in 2000. | | | | | | | | | |
| ^d^ Expected deaths were calculated by multiplying the age-adjusted rate by the total number of populations as presented  in **Additional file 6 table s2**.  ^e^ Including 7 cases of leukocythemia, 3 cases of non-Hodgkin lymphoma, 6 cases of colorectal cancer, 4 cases of  nasopharyngeal carcinoma, 2 cases of bone cancer, 1 case of scalp cancer, 1 case of prostate cancer and 1case of bladder  cancer in the HEA and 4 cases of leukocythemia, 7 cases of colorectal cancer and 7 cases of nasopharyngeal carcinoma  in the LEA. | | | | | | | | | |
