# Supplementary material for: Cancer mortality in a Chinese population surrounding a multi-metal sulphide mine in Guangdong province: an ecologic study
Source: BMC Public Health. 2011 May 16;11:319. doi: 10.1186/1471-2458-11-319 (PMC3112132; doi:10.1186/1471-2458-11-319)
Supplement: Additional file 9 — Mortality data for women from the study regions near the Dabaoshan mine for which the cancer rates (per 100,000) for 2000-2007 as calculated in the present study. The table showed the mortality data for women, including observed deaths, crude rate, age-adjusted rate and expected deaths, from the study regions near the Dabaoshan mine for which the cancer rates for 2000-2007 as calculated of this study. [file 1471-2458-11-319-S9.DOC]

**Table s5 -**Mortality data for women in the study regions near the Dabaoshan mine for which the

cancer rates (per 100,000) for 2000-2007 as calculated in the present study

|  | Village (mortality study region number) | | | | | | | | |
| --- | --- | --- | --- | --- | --- | --- | --- | --- | --- |
| Type of data  (ICD-10) | Shangba  (Ⅰ) | Xiaozhen  (Ⅱ) | Dongfang  (Ⅲ) | Zhongxin  (Ⅳ) | Shaping  (Ⅴ) | Shuikou  (Ⅵ) | Fengshan  (Ⅶ) | Mashan  (Ⅷ) | Madun  (Ⅸ) |
| Heavy metal contami-  nation^a^ | Yes | Yes | Yes | No | No | No | No | No | No |
| All cancer (C00-C97) |  |  |  |  |  |  |  |  |  |
| Observed deaths | 28 | 25 | 20 | 7 | 5 | 4 | 7 | 8 | 9 |
| Crude rate^b^ | 214.51 | 221.10 | 178.17 | 41.09 | 80.96 | 53.05 | 66.03 | 91.19 | 119.81 |
| Age-adjusted rate^c^ | 200.35 | 215.10 | 160.53 | 39.40 | 76.30 | 48.99 | 61.59 | 89.89 | 127.37 |
| Expected deaths^d^ | 26.15 | 24.32 | 18.02 | 6.70 | 4.71 | 3.69 | 6.53 | 7.89 | 9.57 |
| Esophagus cancer (C15) |  |  |  |  |  |  |  |  |  |
| Observed deaths | 9 | 3 | 1 | 2 | 0 | 3 | 1 | 1 | 1 |
| Crude rate^b^ | 68.95 | 26.53 | 8.91 | 11.74 | 0 | 39.79 | 9.43 | 11.40 | 13.31 |
| Age-adjusted rate^c^ | 61.03 | 24.07 | 8.21 | 9.20 | 0 | 37.45 | 9.32 | 11.34 | 15.66 |
| Expected deaths^d^ | 8.88 | 2.72 | 0.92 | 1.57 | 0 | 2.82 | 0.99 | 0.99 | 1.18 |
| Stomach cancer (C16) |  |  |  |  |  |  |  |  |  |
| Observed deaths | 8 | 6 | 14 | 0 | 0 | 0 | 1 | 1 | 0 |
| Crude rate^b^ | 61.29 | 53.06 | 124.72 | 0 | 0 | 0 | 9.43 | 11.40 | 0 |
| Age-adjusted rate^c^ | 56.37 | 47.88 | 114.81 | 0 | 0 | 0 | 11.10 | 10.88 | 0 |
| Expected deaths^d^ | 7.36 | 5.41 | 12.89 | 0 | 0 | 0 | 1.18 | 0.95 | 0 |
| Liver cancer (C22) |  |  |  |  |  |  |  |  |  |
| Observed deaths | 3 | 5 | 3 | 1 | 3 | 1 | 1 | 1 | 4 |
| Crude rate^b^ | 22.98 | 44.22 | 26.73 | 5.87 | 48.59 | 13.26 | 9.43 | 11.40 | 53.25 |
| Age-adjusted rate^c^ | 24.14 | 46.31 | 23.36 | 5.11 | 42.29 | 11.54 | 9.00 | 13.41 | 54.32 |
| Expected deaths^d^ | 3.15 | 5.24 | 2.62 | 0.87 | 2.61 | 0.87 | 0.95 | 1.17 | 4.08 |
| Lung cancer (C33-C34) |  |  |  |  |  |  |  |  |  |
| Observed deaths | 6 | 5 | 1 | 2 | 0 | 0 | 1 | 1 | 1 |
| Crude rate^b^ | 45.97 | 44.22 | 8.91 | 11.74 | 0 | 0 | 9.43 | 11.40 | 13.31 |
| Age-adjusted rate^c^ | 44.84 | 43.51 | 8.50 | 10.95 | 0 | 0 | 8.69 | 10.88 | 12.40 |
| Expected deaths^d^ | 5.85 | 4.92 | 0.95 | 1.86 | 0 | 0 | 0.92 | 0.95 | 0.93 |
| Other cancers^e^ |  |  |  |  |  |  |  |  |  |
| Observed deaths | 2 | 6 | 1 | 2 | 2 | 0 | 3 | 4 | 3 |
| Crude rate^b^ | 15.32 | 53.06 | 8.91 | 11.74 | 32.39 | 0 | 28.30 | 45.59 | 39.94 |
| Age-adjusted rate^c^ | 14.30 | 52.18 | 8.87 | 12.07 | 31.58 | 0 | 27.07 | 43.20 | 38.74 |
| Expected deaths^d^ | 1.87 | 5.90 | 1.00 | 2.05 | 1.95 | 0 | 2.87 | 3.79 | 2.91 |
| ^a^ Based on exposure levels monitored in the 9 villages in 2006 (see **Table 1-2**). | | | | | | | | | |
| ^b^ Numbers in these rows were calculated by respectively dividing the observed deaths by the total number of men or women or  both as presented in **Additional file 6 table s2**. | | | | | | | | | |
| ^c^ Adjusted to China’s age distribution in 2000. | | | | | | | | | |
| ^d^ Expected deaths were calculated by multiplying the age-adjusted rate by the total number of popualrions as presented  in **Additional file 6 table s2**.  ^e^Including 1 cases of leukocythemia, 1 cases of non-Hodgkin lymphoma, 1 case of nasopharyngeal carcinoma, 3 cases of  breast cancer, 1 case of cervix cancer and 2 cases of bone cancer in the HEA and 4 cases of leukocythemia, 5 cases  of nasopharyngeal carcinoma, 3 cases of breast cancer and 2 cases of brain cancer in the LEA. | | | | | | | | | |
